# Identification of renal ischemia reperfusion injury subtypes and predictive strategies for delayed graft function and graft survival based on neutrophil extracellular trap-related genes

**DOI:** 10.3389/fimmu.2022.1047367

**Published:** 2022-12-01

**Authors:** Jiyue Wu, Feilong Zhang, Xiang Zheng, Jiandong Zhang, Peng Cao, Zejia Sun, Wei Wang

**Affiliations:** ^1^ Department of Urology, Beijing Chaoyang Hospital, Capital Medical University, Beijing, China; ^2^ Institute of Urology, Capital Medical University, Beijing, China

**Keywords:** neutrophil extracellular traps, ischemia reperfusion injury, renal transplantation, delayed graft function, graft failure, acute rejection

## Abstract

**Background:**

Ischemia reperfusion injury (IRI) is an inevitable process in renal transplantation, which is closely related to serious postoperative complications such as delayed graft function (DGF), acute rejection and graft failure. Neutrophil extracellular traps (NETs) are extracellular DNA structures decorated with various protein substances released by neutrophils under strong signal stimulation. Recently, NETs have been found to play an important role in the process of IRI. This study aimed to comprehensively analyze the expression landscape of NET-related genes (NRGs) during IRI, identify clusters with different degrees of IRI and construct robust DGF and long-term graft survival predictive strategies.

**Methods:**

The microarray and RNA-seq datasets were obtained from the GEO database. Differentially expressed NRGs (DE-NRGs) were identified by the differential expression analysis, and the NMF algorithm was used to conduct a cluster analysis of IRI samples. Machine learning algorithms were performed to screen DGF-related hub NRGs, and DGF and long-term graft survival predictive strategies were constructed based on these hub NRGs. Finally, we verified the expression of Cxcl1 and its effect on IRI and NETs generation in the mouse IRI model.

**Results:**

This study revealed two IRI clusters (C1 and C2 clusters) with different molecular features and clinical characteristics. Cluster C1 was characterized by active metabolism, mild inflammation and lower incidence of DGF, while Cluster C2 was inflammation activated subtype with a higher incidence of DGF. Besides, based on DGF-related hub NRGs, we successfully constructed robust DGF and long-term graft survival predictive strategies. The mouse renal IRI model verified that Cxcl1 was significantly upregulated in renal tissues after IRI, and using a CXCL8/CXCL1 inhibitor could significantly improve renal function, alleviate renal tubular necrosis, tissue inflammatory response, and NET formation.

**Conclusion:**

This study identified two distinct IRI clusters based on DE-NRGs and constructed robust prediction methods for DGF and graft survival, which can provide references for early prevention and individualized treatment of various postoperative complications after renal transplantation.

## Introduction

Renal transplantation is the most beneficial renal replacement therapy for the treatment of end-stage renal disease (1–3). However, due to the shortage of renal grafts, most patients still endure dialysis treatment for many years. This phenomenon makes it imperative to improve the success rate and long-term survival of allogeneic renal transplantation. Ischemia reperfusion injury (IRI) is a non-negligible procedure in renal transplantation, and it is a major clinical challenge for clinicians in the perioperative period of renal transplantation (4). During I/R of the transplanted kidney, innate immune cells are activated, which secrete multiple inflammatory mediators and induce oxidative stress, causing sterile inflammation injury to endothelial cells and renal tubular epithelial cells, ultimately leading to graft dysfunction (5, 6). Delayed graft function (DGF) is an IRI-related common early complication, which is related to higher rejection rate and poorer short- and long-term outcomes after transplantation (7, 8). Irish et al. (9) proposed a model to predict DGF by integrating multiple risk factors based on data from deceased donor renal transplantation. However, the actual accuracy of this prediction model is not satisfactory, and there is still a lack of effective DGF prediction tools in clinical practice. Thus, it is urgent to have a deeper understanding of the molecular biological changes involved in the process of renal IRI and to develop a new model to better predict the occurrence of DGF.

In response to strong signals, neutrophils release extracellular DNA structures decorated with various protein substances, called neutrophil extracellular traps (NETs) (10). Initially, NET formation has been recognized as a unique mechanism of host defense and pathogen destruction (11, 12). However, with the deepening of research, the role of NETs in sterile inflammation, especially IRI, has attracted widespread attention in recent years (13). In the kidneys, NET formation is a major driver of the self-amplifying cycle of tissue necrosis and inflammation (14). There is a close relationship between renal IRI and NET formation, which promote each other to aggravate the renal necroinflammatory response (15). Besides, the role of NET-related genes (NRGs) in renal IRI has been reported. Raup-Konsavage et al. (16) found that neutrophil PAD4 plays a key role in NET formation during renal IRI. PAD4-deficient mice do not form NETs during renal I/R, and their renal function is restored 48 h following renal I/R (16). Purinergic receptor P2X 1 (P2RX1) was significantly upregulated in kidneys with IRI. P2RX1 supported the formation of NETs following renal IRI, and these NETs were essential for the impairment of mitochondrial dynamics (17). However, a comprehensive and integrated exploration of genes associated with NETs in renal IRI is still lacking.

This study was designed to comprehensively analyze the relationship between NRGs and renal IRI, and to identify renal transplant recipients with different degrees of IRI based on NRGs. In addition, we construct a predictive model for DGF and long-term renal transplant outcomes. We first screened out the differentially expressed NRGs (DE-NRGs) in IRI patients through GEO database and our previous summarized NRGs. The NMF clustering method based on DE-NRGs can divide IRI patients into two clusters with different molecular and clinical characteristics. Besides, we identify the hub genes associated with DGF after renal transplantation by various machine learning methods, and constructed a robust model for the prediction of DGF and long-term renal transplant outcomes after transplantation based on the hub genes. Finally, we also performed experimental validation in the mouse IRI model. It is worth noting that this study is designed to stratify patients and construct multiple prognostic models based on NRGs expression profiles in renal IRI for the first time.

## Materials and methods

### Data collection and processing

The microarray and RNA-seq datasets analyzed in this study were downloaded from the GEO database. Five datasets were finally included and Table 1 shows the relevant information of these datasets. Using matching platform files to obtain gene symbols of each probe matrix. All microarray datasets were normalized through the “limma” R package (18), and Log2 transformation was conducted for subsequent analysis. The NRGs included in this study were collected from previous studies (**Table S1**).

### Identification of DE-NRGs

The GSE43974 dataset contains 203 renal biopsy samples after ischemia-reperfusion injury (IRI) and 188 control samples. Differential expression analysis was performed on the above two types of samples using the “limma” package to identify differentially expressed genes (DEGs). Adjusted p-value< 0.05 and |logFC| > 0.5 were set as the threshold. By intersecting DEGs with NRGs, we finally obtained DE-NRGs between IRI samples and control samples.

### Non-negative matrix factorization algorithm

As renal transplant recipients who have experienced different degrees of IRI may have quite different postoperative graft function and long-term graft survival. To explore the degrees of IRI among different recipients, we conducted a cluster analysis of all IRI samples based on the expression of DE-NRGs using the “NMF” R package to explore potential molecular subtypes (19). The “ brunet “ criterion was selected and iterated 100 times. The number of clusters (k) was set from 2 to 10, and the minimum members of each cluster were set to 2s. The average contour width of the common membership matrix was determined by the R package “NMF”. Using the cophenetic correlation coefficients (from 0 to 1) to reflect the stability of clusters, while the residual sum of squares (RSS) was used to reflect the model’s clustering performance. The optimal k was selected based on the cophenetic, dispersion and silhouette metrics. Through the above algorithm and the optimal k, IRI samples are divided into different molecular clusters.

### Identification of DGF-related hub genes by machine learning methods

To construct a predictive model for DGF after renal transplantation, we performed two machine learning methods to screen DGF-related hub genes. The random forest (RF) algorithm is a supervised classification method based on an ensemble of decision trees, which can be implemented by the “randomForest” R package and ranks features according to the Gini importance measure (20). The support vector machine recursive feature elimination (SVM-RFE, based on the “e1070” R package) algorithm is a recursive feature elimination strategy that uses the weighted vectors generated from the SVM to optimize the classification accuracy between different groups (21). By intersecting the top-ranked genes from the RF algorithm and genes obtained by the SVM-RFE algorithm, we finally identified eight DGF-related hub NRGs.

### Establishment and validation of the DGF predictive model

IRI samples in the GSE43974 dataset were randomly divided into a training set and an internal testing set (1: 1), and then performed a least absolute shrinkage and selection operator (LASSO) regression analysis with 10-fold cross-validation on these DGF-related hub NRGs using the “glmnet” R package to screen candidate NRGs (22). Linearly combining the regression coefficient obtained and expression of each candidate NRGs to calculate the risk score:


$$Risk\;score=\sum_{i=1}^{n}({\text{coef}}_{i\;}\times {\text{Exp}}_{i})\;$$

IRI samples were stratified into two groups (the high-risk and the low-risk group) according to the median risk score. The accuracy of our model was assessed through the receiver operating characteristic (ROC) curves and a 10-fold cross-validation. Besides, an external validation set (GSE37838) was used to validate the robustness of the predictive model and to compare the model with several other predictive strategies.

### Establishment of a predictive signature for the long-term survival of renal allograft

Studies have shown that the DGF after renal transplantation is significantly related to the occurrence of acute rejection (AR) and long-term graft failure. Based on the DGF-related hub NRGs, we sought to establish a predictive signature of long-term graft failure in the GSE21374 dataset. Renal transplant recipients were randomly divided into a training set and a validation set at a ratio of 1:1. Firstly, using the univariable Cox regression (HR ≠ 1 and p< 0.05) to screen out prognosis-related NRGs, and then a LASSO regression algorithm with 10-fold cross-validation was used to establish the final predictive signature. Exporting the coefficient value of each gene from the LASSO regression algorithm, and the risk was calculated using the expression of each NRG and their corresponding regression coefficient. Renal transplant recipients were also divided into the high-risk group and the low-risk group based on the median risk. Kaplan–Meier (K–M) survival curves were used to compare the survival of different groups, and the time‐dependent receiver operating characteristic (ROC) curves were used to measure the predictive performance of our signature (23).

### Functional enrichment analysis

Gene Ontology (GO) and Kyoto Encyclopedia of Genes and Genomes (KEGG) pathway enrichment analyses were conducted using the “clusterprofiler” R package (24). Gene Set Enrichment Analysis (GSEA) was used to compare biological processes that were significantly different between IRI groups. The activity scores of specific biological pathways in each IRI sample were assessed by single sample Gene Set Enrichment Analysis (ssGSEA) using the “GSVA” R package, and the reference gene sets included in the specific biological pathways were obtained from the MSiDB database (25). Terms with< 0.05 and FDR (q-value)< 0.25 were statistically significant.

### Calculation of immune cell infiltration

Using the ssGSEA to measure the infiltration of 23 types of immune cells between different IRI groups, and the pod-plot was used to compare immune cell infiltration. Besides, we also validated the infiltration of neutrophils by other five algorithms (TIMER, CIBESORT, CIBESORT-ABS, QUANRTISEQ, and MCPcounter).

### Mice and renal IRI model

Male, 6-8 weeks old C57BL/6 N mice (Weitong Lihua Experimental Animal Center, Beijing, China) were used in this study, and all mice were maintained in a specific pathogen-free environment. All mice were starved for 12 hours before the operation. The mice were anesthetized with an intraperitoneal injection of pentobarbital (60 mg/kg), and the core body temperature was maintained between 34 and 36°C. The right kidney was removed from the mouse, and the blood flow was blocked at the left renal pedicle using a noninvasive sterile vascular clamp for 35 minutes, then the clamp was released for renal reperfusion. Muscle and skin were sequentially closed with 5-0 silk thread as previously reported (26). One milliliter of normal saline was injected intraperitoneally to prevent dehydration. Sham-operated mice had the right kidney removed and the left renal tip exposed without clamping. The G31P-treated group was administrated intraperitoneally with G31P at a dose of 0.5 mg/kg once every 2 days before ischemia. Mice were euthanized 24 hours after renal IRI, and kidney tissues and serum were collected for subsequent analysis.

### Assessment of kidney injury and renal function

Kidney tissues were soaked in 10% formalin and then dehydrated and hyalinized using alcohol and xylene, respectively. Using paraffin to embed the kidney tissues and then slicing them into thin sections (4μm), then the sections were fully dewaxed with xylene and hydrated by gradient alcohol for hematoxylin and eosin (H&E) staining. Separating serum from whole blood samples and measuring the levels of serum creatinine and BUN using the creatinine detection kit (Abcam) and urea detection kit (Abcam) according to the manufacturer’s instructions.

### Quantitative real-time PCR

Using TRIzol reagent (#15596026, Thermo Fisher Scientific) to isolate and extract total RNA, and the reverse transcription was conducted using a PrimeScript™ RT Reagent Kit (#RR600A, TaKaRa Bio). Then, according to the TB Green Premix Ex Taq (TaKaRa Bio, Inc.) protocol, using specific primers to perform quantitative real-time PCR. GAPDH wasused as an internal control and the 2^-ΔΔCt^ method was used to calculate relative mRNA levels. The primers sequences of the Cxcl1 in this study are as follows: Forward: *5’-CTGGGATTCACCTCAAGAACATC-3’*, Reverse: *5’-CAGGGTCAAGGCAAGCCTC-3’*.

### Assessment of tissue inflammation and detection of serum NETs

Paraffin-embedded kidney tissues were dewaxed in xylene and then hydrated with graded concentrations of ethanol. Using 3% hydrogen peroxide solution to block endogenous peroxidase for 10 minutes and using citrate buffer to perform antigen retrieval. After blocking with 10% goat serum for 30 minutes, the rabbit polyclonal anti-Neutrophil Elastase (anti-NE) primary antibody (Abcam, ab68672) diluted at a ratio of 1:100 was used to incubate overnight at 4°C, and then HRP-conjugated secondary antibody was used to incubate for immunohistochemical (IHC) staining. To quantify the production of NETs, we assessed the expression of dsDNA, NE and MPO in mouse serum. The levels of dsDNA and NE were measured by mouse anti-double stranded DNA antibody (dsDNA) ELISA kits (Wuhan ColorfulGene Biological Technology Co., LTD, JYM1061Mo) and mouse neutrophil elastase ELISA kits (Wuhan ColorfulGene Biological Technology Co., LTD, JYM0280Mo) respectively according to the manufacturer’s directions. While the serum MPO was detected by using the MPO assay kit (colorimetric method).

### Statistical analysis

The statistical analysis involved in this study was performed by R software (version 4.2.1). Descriptive statistics were used to characterize the distributions of continuous variables (mean, median, quartiles, range, standard error, standard error of mean) and nominal variables (frequency, percentage). For normal distribution variables, Student’s t-test was used to compare the differences between the two groups, while Mann-Whitney U test was used for abnormally distributed variables. The Chi-square test was used to analyze the relationship among IRI groups and Donor type, DGF, AR. All tests were two-sided and p< 0.05 was considered statistically significant.

## Results

### Identification of DE-NRGs and functional enrichment analysis

**Figure 1:**
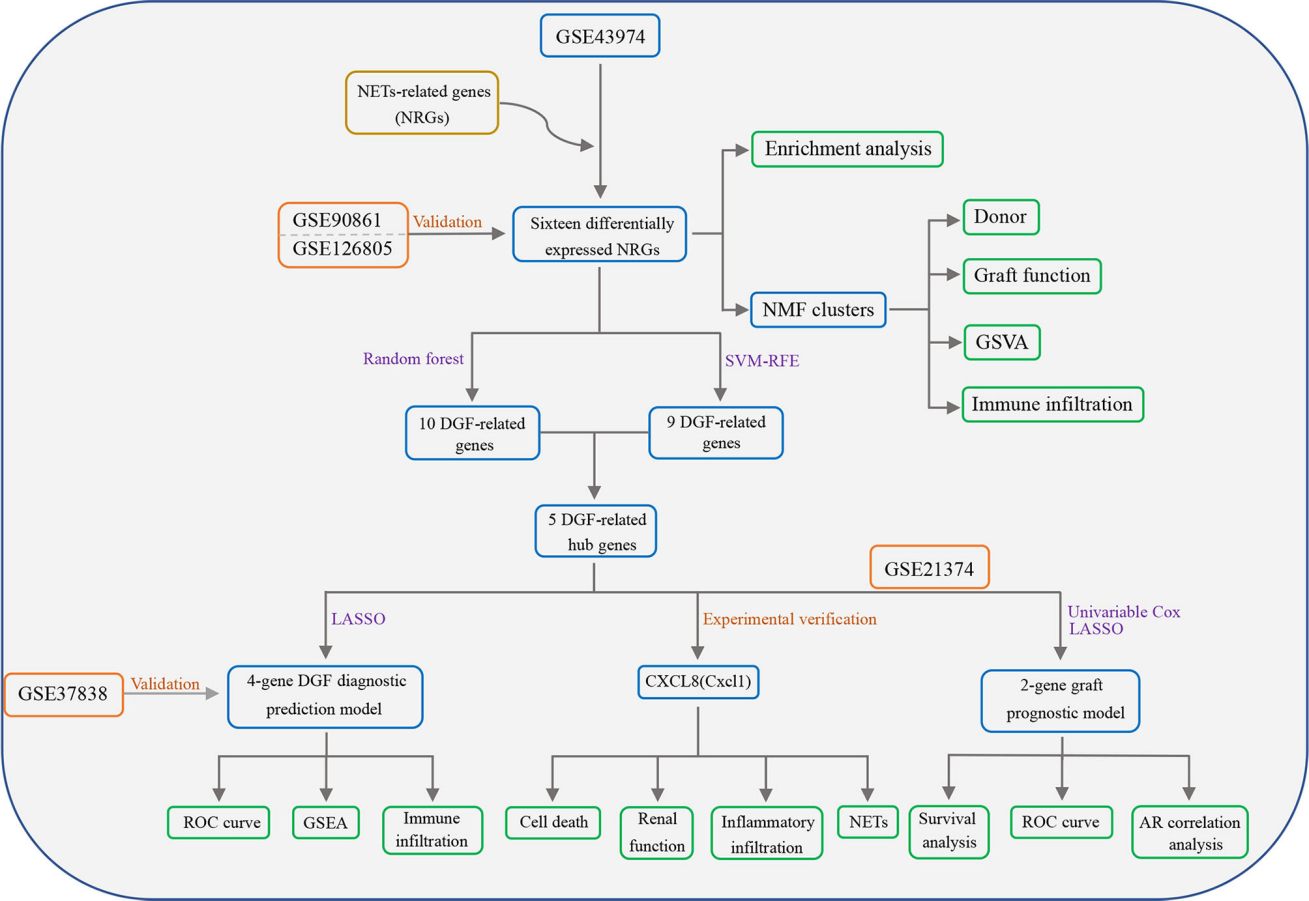
Flowchart of this study. NETs, Neutrophil extracellular traps; NRGs, NET-related genes; DGF, delayed graft function; GSVA, gene set variation analysis; SVM-RFE, support vector machine recursive feature elimination; LASSO, least absolute shrinkage and selection operator; ROC, receiver operating characteristic; GSEA, gene set enrichment analysis; AR, acute rejection.

**Figure 2:**
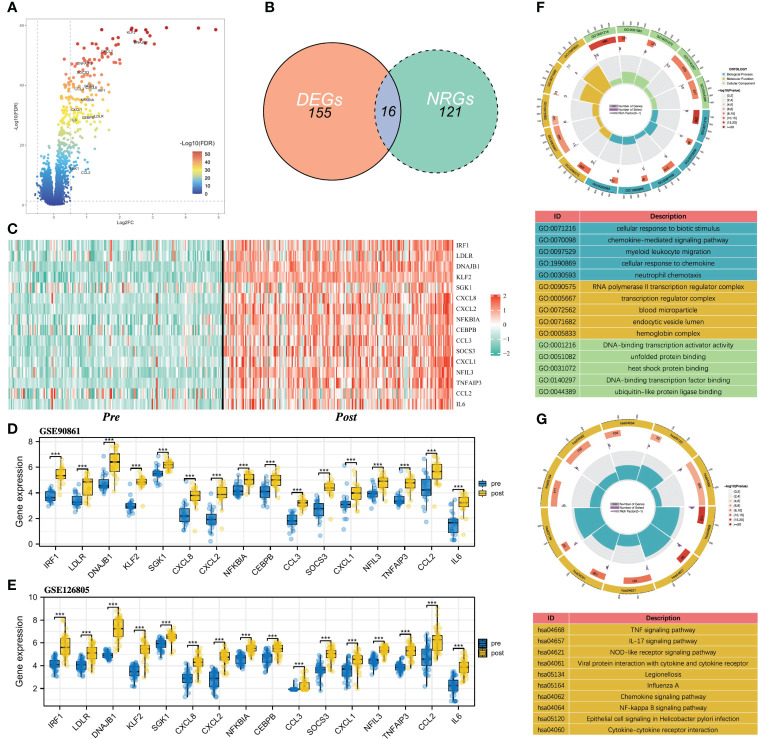
Identification of DE-NRGs and functional enrichment analysis. **(A)** Volcano plot of DEGs, and the gene symbols of DE-NRGs were labeled. **(B)** Intersection between DEGs and NRGs in renal IRI. **(C)** Heatmap of the expression of 16 DE-NRGs in control samples and IRI samples. **(D, E)** Box and scatter plots showing the expression of 16 upregulated DE-NRGs in GSE90861 and GSE126805. **(F)** GO enrichment analysis of DE-NRGs in terms of biological process, cellular component, and molecular function. **(G)** KEGG pathway analysis of DE-NRGs. NRGs, NET-related genes; DEGs, differentially expressed genes; DE-NRGs, differentially expressed NRGs; IRI, ischemia reperfusion injury; GO, Gene Ontology; KEGG, Kyoto Encyclopedia of Genes and Genomes. ***P < 0.001.

**Figure 1** shows the flowchart of our study. Differential expression analysis was performed on the gene expression profiles of 188 control samples and 203 IRI samples. According to the conditions described in the “Materials and Methods” section, a total of 171 DEGs were identified and all of them were significantly upregulated in the IRI samples (**Figure 2A**). By integrating 171 DEGs with the 137 NRGs we collected, 16 DE-NRGs were finally obtained (**Figure 2B**, **Table S2**). **Figure 2C** showed the expression landscape of the 16 DE-NRGs in all control and IRI samples, which can be seen that all DE-NRGs were significantly upregulated in allograft kidney tissues after IRI. To further determine the expression of 16 DE-NRGs after IRI, we used two independent renal transplant cohorts for validation. As shown in **Figures 2D, E**, these DE-NRGs were significantly upregulated in allograft kidney tissues after IRI.

To gain insight into the potential role of DE-NRGs in IRI, we performed a functional enrichment analysis on DE-NEGs. GO analysis showed that DE-NRGs are related to biological processes such as chemokine-mediated signaling pathway, myeloid leukocyte migration, cellular response to chemokine, and neutrophil chemotaxis (**Figure 2F**). Besides, the KEGG results showed that TNF signaling pathway, IL-17 signaling pathway, NF-kappa B signaling pathway, Chemokine signaling pathway, and NOD-like receptor signaling pathway were significantly enriched (**Figure 2G**).

### Stratification of IRI patients based on DE-NRGs

**Figure 3:**
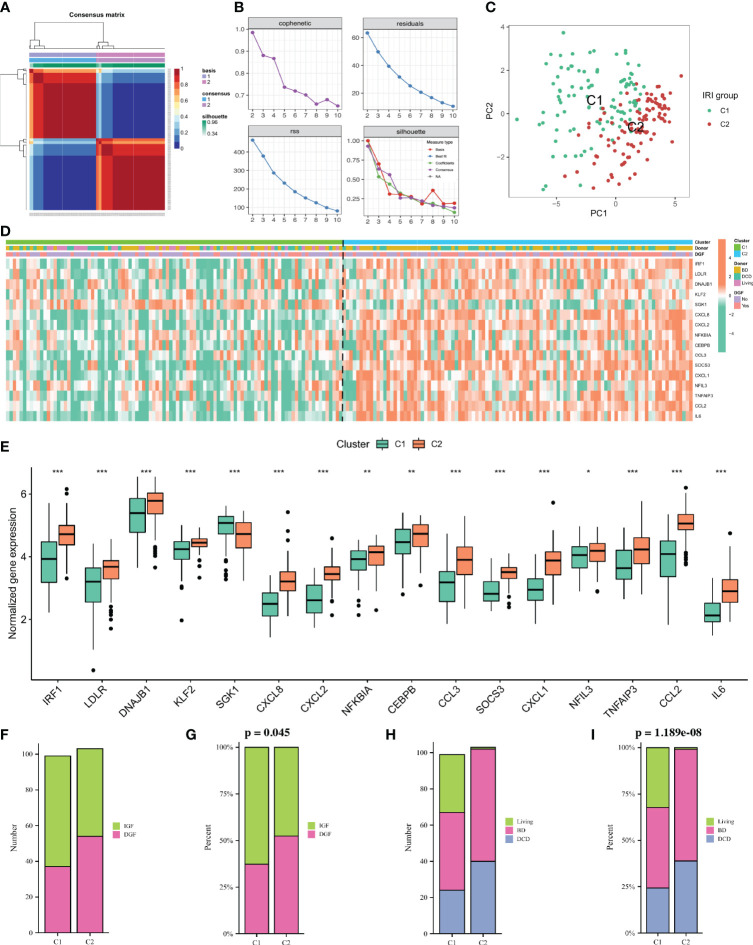
Non-negative matrix factorization (NMF) analysis for the IRI samples. **(A)** Consensus map of NMF clustering when k = 2. **(B)** Distribution of cophenetic, residuals, RSS and silhouette with a rank of 2–10. **(C)** A PCA plot of the expression profile of DE-NRGs between two clusters. **(D)**: Heatmap showing the expression landscape of DE-NRGs and the clinical characteristics of the two clusters. **(E)**: Box plot showing the expression of 16 DE-NRGs between the two clusters. **(F, G)**: Histogram comparing the differences in donor between the two clusters. **(H, I)** Histogram comparing the differences in the occurrence of DGF between the two clusters. IRI, ischemia reperfusion injury; RSS, residual sum of squares; PCA, principal component analysis; DE-NRGs, differentially expressed NET-related genes; DGF, delayed graft function. *P < 0.05, **P < 0.01, ***P < 0.001.

To identify kidney transplant recipients with different degrees of IRI, we performed cluster analysis using the NMF algorithm based on the expression profiles of 16 DE-NRGs in all IRI samples. Based on the cophenetic, dispersion and silhouette metrics, k = 2 was ultimately determined as the optimal cluster number (**Figures 3A, B**). Therefore, according to the NMF algorithm, all IRI samples were divided into two clusters, namely the C1 cluster (n = 100) and the C2 cluster (n = 103). Principal component analysis (PCA) showed that the expressions of DE-NRGs between the two IRI clusters (C1 vs C2) were significantly different (**Figure 3C**). **Figure 3D** showed the expression landscape of DE-NRGs and the clinical characteristics of kidney transplant recipients between the two IRI clusters. Specifically, except for SGK1, which was upregulated in the C1 cluster, the rest of the DE-NRGs were all upregulated in the C2 cluster (**Figure 3E**). As for clinical characteristics, the percentage of DGF in the C2 cluster was higher than that in the C1 cluster (p = 0.045) (**Figures 3F, G**). Additionally, the donors of the C2 cluster were more obtained from BD and DCD patients, while the donors of the C1 cluster were mainly obtained from living patients (p< 0.001; **Figures 3H, I**).

### GSVA enrichment analysis and immune infiltration analysis of IRI clusters

**Figure 4:**
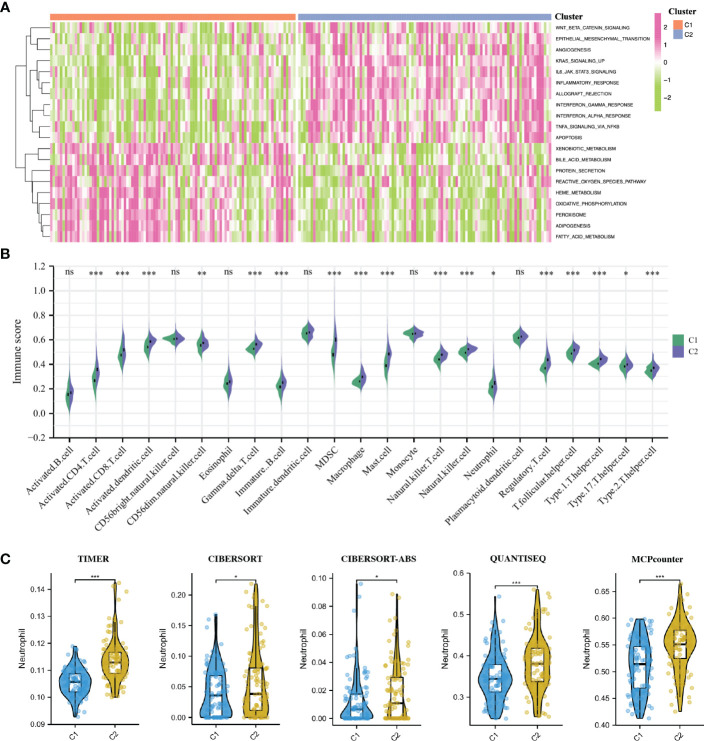
GSVA enrichment analysis and immune infiltration analysis of IRI clusters. **(A)**: The heatmap showing the different hallmarks between the two clusters. **(B)** Pod-plot showing the infiltration of 23 types of immune cells between the two clusters. **(C)** Comparison of the infiltration of neutrophils between clusters using other five algorithms. GSVA, gene set variation analysis. *P < 0.05, **P < 0.01, ***P < 0.001; ns, no significance.

To elucidate the different biological characteristics between the two IRI clusters, we performed GSVA enrichment analysis based on the Hallmarks gene set (h.all.v7.2.symbols.gmt) from the MSigDB database. The heatmap showed that the KRAS signaling up, IL6-JAK-STAT3 signaling, interferon α/β response, inflammatory response, apoptosis and allograft rejection were significantly activated in the C2 cluster. While some metabolic-related processes such as fatty acid metabolism, heme metabolism, bile acid metabolism, protein secretion and oxidative phosphorylation were activated in the C1 cluster (**Figure 4A**).

The result of immune infiltration analysis showed that a variety of immune-related cells including B cells, CD8^+^ T cells, CD4^+^ T cells, NK cells, dendritic cells, macrophages and neutrophils had higher abundance in the C2 cluster compared with that in the C1 cluster (**Figure 4B**). Furthermore, through five other algorithms, we further confirmed that the abundance of neutrophils in the C2 cluster was significantly higher (**Figure 4C**). Suggesting that the inflammatory and immune responses in the C2 cluster were more severe, the production of NETs in these renal grafts may be more, and the postoperative graft function and long-term graft survival may be worse.

### Construction and validation of DGF predictive model

**Figure 5:**
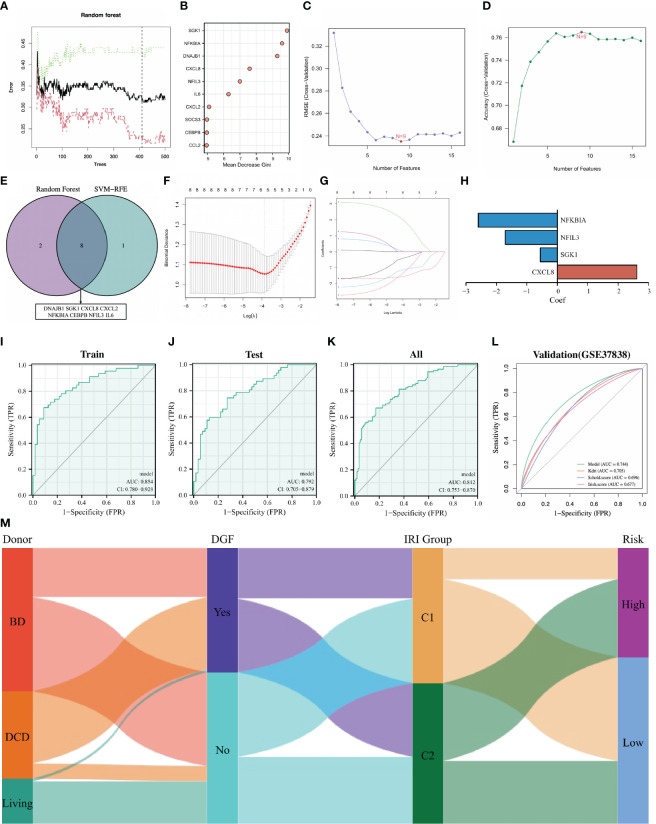
Establishment and validation of the DGF predictive model. **(A)** Random forest tree. The abscissa represents trees and the ordinate represents the error rate. Red represents the DGF samples, green represents the non-DGF samples, and black represents the overall samples. The dotted line represents the tree holding the minimum error rate. **(B)**: Gini importance measure. The horizontal axis represents mean decrease Gini, and the vertical axis represents characteristic NRGs. **(C, D)** Feature NRGs were selected with the SVM-RFE algorithm at the optimal point. **(E)**: Intersecting the top 10 NRGs identified by the RF and the 9 NRGs screened by the SVM-RFE. **(F, G)**: The 4 candidate NRGs obtained by LASSO regression with 10-fold cross-validation. **(H)**: LASSO coefficients profiles of candidate NRGs in the model. **(I–L)**: Evaluating the performance of the model in the training set, internal testing set, whole set and external validation set using ROC curves. **(M)**: Sankey diagram showing the relationships among the type of donors, the occurrence of DGF, the IRI cluster and the risk of IRI samples. DGF, delayed graft function; NRGs, NET-related genes; SVM-RFE, support vector machine recursive feature elimination; RF, random forest; LASSO, least absolute shrinkage and selection operator; ROC, receiver operating characteristic; IRI, ischemia reperfusion injury.

Delayed graft function (DGF) is the earliest and most important complication of IRI, developing an effective prediction strategy for DGF is a research hotspot in the field of renal transplantation. Since DE-NRGs are differentially expressed in renal tissues with different degrees of IRI and renal grafts classified by 16 DE-NRGs have large differences in the incidence of DGF, we attempted to construct a robust predictive model for DGF based on DE-NRGs. DGF-related feature NRGs were identified through the RF algorithm, and 16 DE-NRGs were ranked according to the Gini importance measure (**Figures 5A, B**). The SVM-RFE algorithm was also used to screen DGF-related feature NRGs, and the number of NRGs was determined according to the smallest error (highest accuracy) (**Figures 5C, D**). By intersecting the top 10 NRGs identified by the RF algorithm and the 9 NRGs screened by the SVM-RFE algorithm, we finally obtained 8 DGF-related hub NRGs (**Figure 5E**; **Table S3**).

We randomly divided the 203 IRI samples into a training set and an internal testing set in a 1:1 ratio. Then, the candidate NRGs (NFKBIA, NFIL3, SGK1 and CXCL8) were selected for constructing the DGF predictive model by LASSO regression with their regression coefficients as -2.598, -1.706, -0.548 and 2.622, respectively (**Figures 5F–H**). The risk score was calculated for each IRI sample and all samples were divided into high-risk and low-risk groups according to the median risk score. The ROC curve was used to assess the accuracy of the predictive model, and the results showed that the area under the curve (AUC) for the training set, the internal testing set, and the whole set was 0.854, 0.792, and 0.812, respectively (**Figures 5I–K**). To further validate the universality and robustness of our predictive model, we performed a 10-fold cross-validation of the model in the whole set (**Table S4**). The result showed that the average sensitivity is 0.83, the average specificity is 0.67 and the average AUC is 0.75. Besides, an external test set (GSE37838) was also used to further validate tour model, and the AUC of this set was 0.744 (**Figure 5L**). It was worth noting that in the GSE3783 dataset, the prognostic performance of our models was better than other traditional DGF predictive methods (Kdri model, Schol score and Irish score) (**Figure 5L**). Overall, our DGF predictive model based on the expression of 4 NRGs was satisfactory. The Sankey diagram intuitively showed the relationships among the type of donors, the occurrence of DGF, the IRI cluster and the risk of IRI samples (**Figure 5M**).

### Function enrichment analysis and immune infiltration analysis based on DGF predictive model

**Figure 6:**
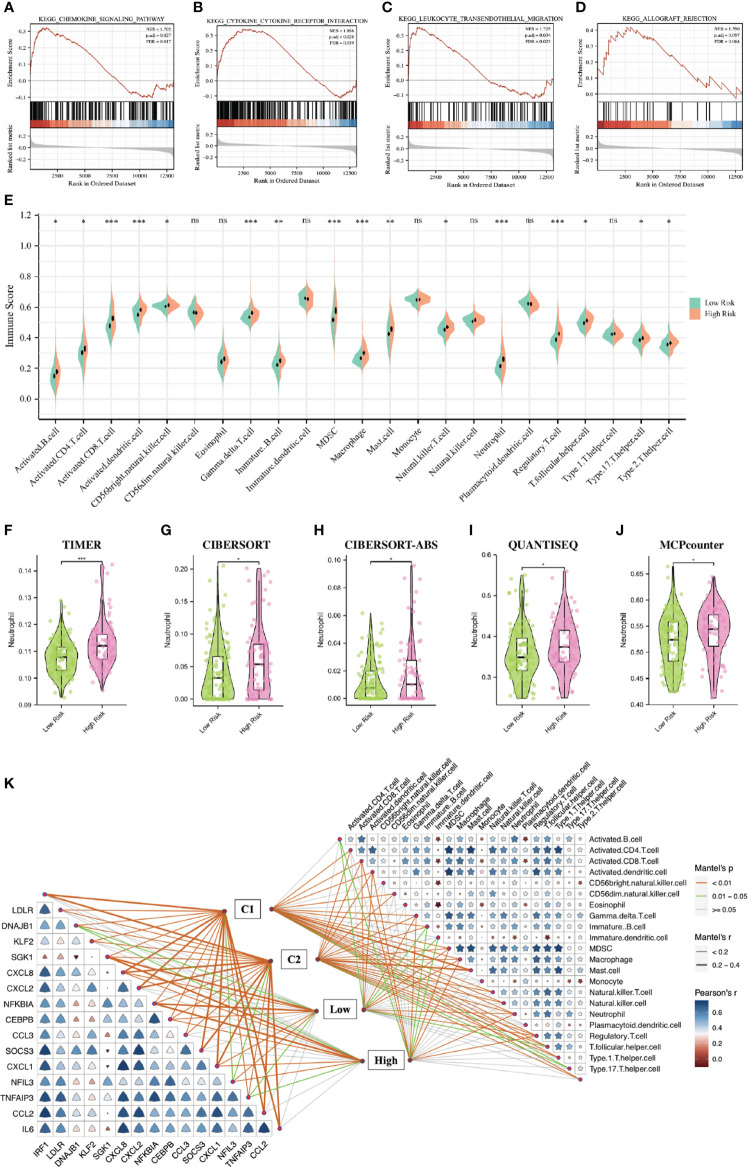
Function enrichment analysis and immune infiltration analysis based on the DGF predictive model. **(A–D)**: GSEA showing the different pathways between the two IRI groups. **(E)**: Pod-plot showing the infiltration of 23 types of immune cells between the two groups. **(F–J)**: Comparison of the infiltration of neutrophils between groups using other five algorithms. **(K)**: the correlation between 23 immune-related cells, the correlation between 16 DE-NRGs and the relationships between immune-related cells, DE-NRGs and IRI subgroups classified by two strategies. DGF, delayed graft function; GSEA, gene set enrichment analysis; IRI, ischemia reperfusion injury; DE-NRGs, differentially expressed NET-related genes. *P < 0.05, **P < 0.01, ***P < 0.001; ns, no significance.

To explore the underlying biological mechanisms that lead to differences between high- and low-risk groups, we performed GSEA enrichment analysis based on the KEGG gene set (c2.cp.v7.2.symbols.gmt) in the MSigDB database. The results showed that the chemokine signaling pathway, cytokine-cytokine receptor interaction, leukocyte transendothelial migration and allograft rejection were significantly enriched in the high-risk group (**Figures 6A–D**). SsGSEA analysis showed that the abundances of B cells, CD8^+^ T cells, CD4^+^ T cells, NK cells, dendritic cells, macrophages and neutrophils in the high risk group were higher than that in the low risk group (**Figure 6E**). Furthermore, through five other algorithms, we further confirmed that the abundance of neutrophils was significantly higher in the high-risk group (**Figures 6F–J**). **Figure 6K** showed the correlation between 23 immune-related cells, the correlation between 16 DE-NRGs and the relationships between immune-related cells, DE-NRGs and IRI subgroups classified by two strategies.

### Construction and validation of a long-term graft survival predictive signature

In the GSVA analysis and the GSEA analysis (**Figure 4A**; **6D**), we found that the allograft rejection was significantly enriched in both the C2 cluster and the high-risk group. Since the occurrence of DGF after transplantation is closely related to allograft rejection and long-term graft failure, in order to effectively predict the long-term graft survival and the possibility of AR, we aimed to construct a signature for predicting long-term graft survival based on the DGF-related hub NRGs.

**Figure 7:**
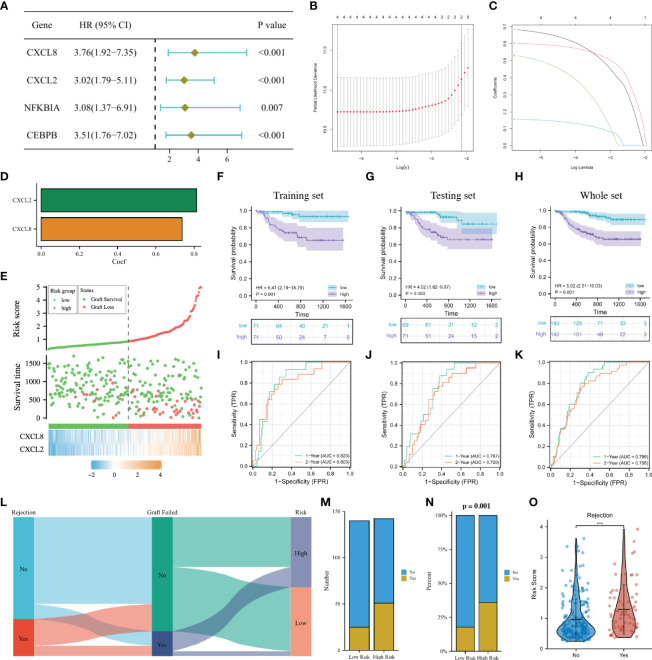
Construction of a predictive signature for the long-term survival. **(A)**: Forrest plot of univariable Cox regression analysis. **(B, C)**: The 2 candidate NRGs obtained by LASSO regression with 10-fold cross-validation. **(D)**: LASSO coefficients profiles of candidate NRGs in the signature. **(E)**: Risk map of the two risk groups. **(F–H)**: K-M survival analysis of the model in the training set, the testing set and the whole set. **(I–K)**: Time-dependent ROC analysis in the training set, the testing set and the whole set. **(L)**: Sankey diagram showing the relationship among the occurrence of AR, long-term graft failure and the risk of recipients. **(M, N)**: Histogram comparing the differences in the occurrence of AR between the two groups. **(O)**: Comparison of the risk score between AR and non-AR recipients. NRGs, NET-related genes; LASSO, least absolute shrinkage and selection operator; K-M, Kaplan–Meier; ROC, receiver operating characteristic; AR, acute rejection.

In the GSE21374 dataset, we randomly divided kidney transplant recipients into a training set and a testing set (1:1). In the training set, a univariable Cox regression analysis was performed on the eight DGF-related hub NRGs and four prognostic NRGs (CXCL8, CXCL2, NFKBIA, and CEBPB) were identified (**Figure 7A**). Then LASSO regression analysis was used to sub-selected NRGs for signature construction, and the risk of each recipient was calculated as follows: Risk = 0.74 * Exp_(CXCL8)_ + 0.81 * Exp_(CXCL2)_ (**Figures 7B–D**). **Figure 7E** showed the expressions of CXCL8 and CXCL2 in the high-risk group were significantly higher, and the recipients with higher risk were more likely to undergo graft failure. K-M curves indicated that high-risk recipients had worse long-term graft survival than low-risk recipients regardless of the cohort (**Figures 7F–H**). The time-dependent ROC curves showed that in the training set, the AUC of the signature at 1 year and 2 years were 0.823 and 0.803, respectively. In the testing set, the AUC at 1 year and 2 years were 0.767 and 0.720, respectively. While in the whole set, the AUC at 1 year and 2 years were 0.795 and 0.758, respectively (**Figures 7I–K**). The above results all showed that our 2-NRG predictive signature can accurately predict the long-term outcome of grafts.

The Sankey diagram intuitively showed the relationship among the occurrence of AR, long-term graft failure and the risk of recipients (**Figure 7L**). Correlation analysis implied that high-risk recipients were more likely to develop AR, and recipients who experienced AR may have a higher risk (**Figures 7M–O**). Early screening of such recipients and timely adjustment of their immunosuppressive therapy may effectively improve the survival of kidney grafts.

### Validate the expression of CXCL8 in IRI and its effect on NETs production

**Figure 8:**
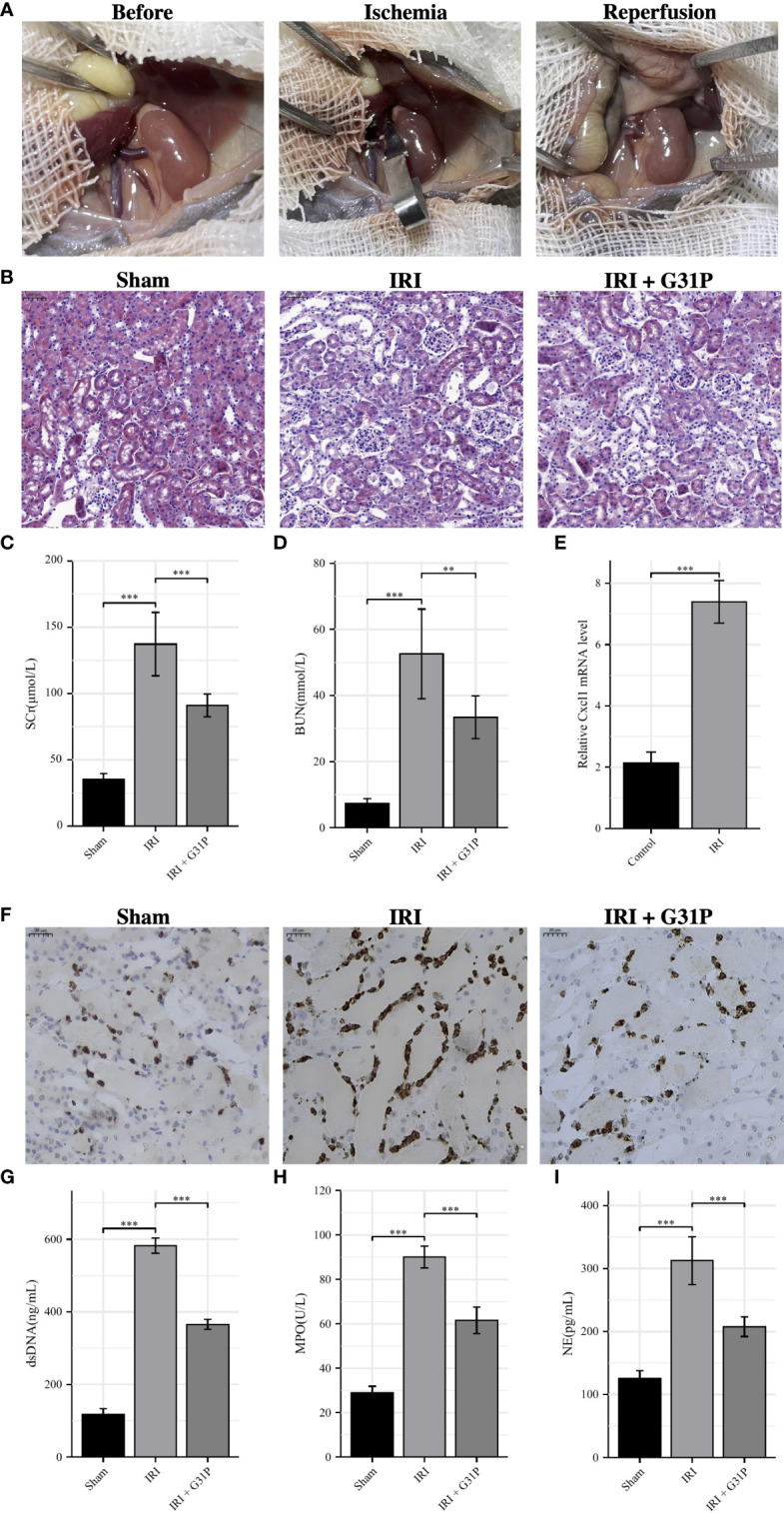
Experimental verification processes. **(A)**: The surgical process of renal ischemia-reperfusion in mouse. **(B)**: H&E staining of the renal tissues from Sham mice, IRI mice and IRI+ G31P mice. **(C, D)**: Serum creatinine and BUN were measured in the Sham group, the IRI group and the IRI+G31P group. **(E)**: mRNA expression of Cxcl1 in kidney tissue of the control group and the IRI group. **(F)**: Immunohistochemistry staining of NE in Sham mice, IRI mice and IRI+ G31P mice. **(G–I)**: NET-related markers (dsDNA, MPO, NE) in the serum of the Sham group, the IRI group and the IRI+G31P group. H&E, hematoxylin and eosin; IRI, ischemia reperfusion injury; NE, neutrophil elastase; MPO, myeloperoxidase. **P < 0.01, ***P < 0.001.

Since CXCL8 was included in both predictive models, we selected it for further experimental validation. Human CXCL8, formerly known as IL-8, is a member of the chemokine superfamily and is closely associated with inflammatory diseases. Its homolog in mice is Cxcl1. G31P is a CXCL8 mutant prepared by SNP mutation, which has a high affinity for binding to CXCR1/CXCR2 but has no biological activity, so it is usually used as an antagonist of CXCL8. We first conducted the mouse IRI model (ischemia for 35 minutes followed by reperfusion for 24 hours), and **Figure 8A** showed the complete surgical process of renal ischemia-reperfusion in mice. Compared with the sham group, the HE staining of the renal tissues of the IRI group showed obvious tubular cell death and effacement of brush border, while pretreatment with G31P could significantly alleviate the IRI (**Figure 8B**). Besides, the IRI group had significantly increased serum creatinine and BUN at 24 h after reperfusion compared with the sham group, and pretreatment with G31P improved renal function (**Figures 8C, D**). After IRI, the mRNA expression of Cxcl1 was validated in mice renal tissues by qRT-PCR, and the results showed that the expression of Cxcl1 after IRI was significantly upregulated compared with the control (p< 0.001; **Figure 8E**). The exploration of neutrophils and NETs showed that the infiltration of neutrophils in mice renal tissues increased significantly after IRI, and pretreatment with G31P could alleviate the infiltration of neutrophils (**Figure 8F**). Besides, NET-related markers (dsDNA, MPO, NE) in the serum of the IRI group were also significantly higher than those in the sham group, while pretreatment with G31P can decrease the production and release of these markers (**Figures 8G–I**). Overall, consistent with the results of bioinformatic analyses, we found that CXCL8/CXCL1 was significantly upregulated in renal tissues after IRI, and inhibiting the expression and function of CXCL8/CXCL1 can reduce the production of NETs and the severity of IRI.

## Discussion

IRI and allograft rejection are two major factors affecting graft survival after renal transplantation. Among them, IRI occurring during renal transplantation causes acute kidney injury and DGF, and may eventually lead to graft loss and transplant failure (4). Therefore, alleviating IRI in renal transplantation help to reduce the incidence of DGF and improve the long-term graft survival. NETs are thought to be web-like structures composed of DNA and granule proteins that are released after cell death (11). NETs are induced during renal I/R, and NET formation aggravates renal injury, which further induces more NET formation. This induces a pro-inflammatory positive feedback loop, which in turn exacerbates renal IRI (14, 15). Currently, there are few studies about NRGs and IRI, and the available studies mainly focused on the relationship between a single NRG and renal IRI. Therefore, this study aimed to comprehensively analyze the relationship between NRGs and renal IRI and its effects on the transplanted kidney (DGF, acute rejection, graft survival). We identified DE-NRGs during renal IRI and clustered renal IRI patients based on the expression profiles of DE-NRGs to identify subclasses with different molecular and clinical characteristics. In addition, we identified the hub genes related to DGF and constructed a predictive model for DGF and long-term outcome of renal transplantation. Finally, we performed validation in the mouse IRI model and found that Cxcl1 (mouse homolog of human CXCL8) (27) was significantly upregulated after IRI. The use of CXCL8/CXCL1 inhibitor significantly reduced NET formation and attenuated renal IRI.

In 2004, neutrophils were first reported to kill pathogens through the formation of NETs (11). Since then, a large number of studies have focused on NETs. In addition to capturing and killing microorganisms causing infectious diseases, NETs are also involved in the occurrence and development of non-infectious diseases, including cancer (28), autoimmune diseases (29, 30), thrombosis (31), and sterile inflammatory tissue injury (32). Among them, renal IRI is a typical type of sterile inflammatory injury in which NETs play an important role (33, 34). Studies have shown that inhibition of NET formation or promotion of NET degradation by PAD4 inhibitor or DNase I can improve renal IRI (16, 35). In this study, we obtained 16 DE-NRGs by integrating 171 DEGs before and after IRI and 137 NRGs, all of which were upregulated in renal tissues after IRI. The results of functional enrichment analysis showed that they were significantly enriched in TNF signaling pathway, IL-17 signaling pathway and NOD-like receptor signaling pathway. Studies have shown that neutrophils from patients with ulcerative colitis can produce NETs upon stimulation with TNF-α, and reduced NET formation and their related proteins can be observed in patients successfully treated with anti-TNF-α therapy (36). In addition, IL-17A aggravates liver injury after I/R by inducing neutrophil infiltration and NET formation (37). NLRP3 inflammasome is also involved in the formation of NETs, which is dependent on PAD4 (32). Inhibition of NLRP3 inflammasome signaling significantly attenuates NET formation in the non-infected state (32). Subsequently, based on the expression profiles of the 16 DE-NRGs in IRI tissues, we divided IRI patients into C1 and C2 groups through NMF clustering analysis. Except for SGK1, the remaining 15 DE-NRGs were significantly upregulated in the C2 group of IRI samples. It was shown that NETs can induce dendritic cells (DCs) activation and promote Th1 polarization in patients with type 1 diabetes (38). In addition, NETs promote macrophage inflammation in diabetic mice (39). This is consistent with our finding of a higher infiltrating abundance of various immune cells such as DCs, macrophages, neutrophils and Th1 in the C2 group of IRI samples. All of these results suggest a higher immune inflammatory response and NET formation in the C2 group of IRI samples and that the prognosis in C2 group patients may be worse.

DGF is a common early complication related to IRI during renal transplantation, which severely affects the short- and long-term survival of the transplanted kidneys (7). We found significant differences in the incidence of postoperative DGF among IRI subgroups clustered according to DE-NRGs. In order to identify patients at high risk of DGF for early intervention, we attempted to construct a DE-NRGs-based model to predict the occurrence of DGF. By integrating machine learning method, we obtained 8 hub genes (hub-NRGs) related to DGF, namely DNAJB1, SGK1, CXCL8, CXCL2, NFKBIA, CEBPB, NFIL3, and IL6. Available studies have shown that these genes are closely linked to NETs. Among them, cigarette smoke can alter neutrophil chemotaxis, NET formation and the expression of inflammatory-related gene DNAJB1 (40). In atherosclerotic cardiovascular diseases, increased intracellular Cl- concentration in neutrophils promotes NET formation *via* Cl–sensitive SGK1 signaling (41). In addition, NFIL3 can aggravate the inflammatory response in gout by stimulating neutrophil autophagy and the formation of NETs through REDD1/mTOR (42). Silencing of NFIL3 reduces inflammatory injury in mice with acute gouty arthritis through inhibiting neutrophil autophagy and the formation of NETs (42). Studies have reported that low concentrations of NETs induce proliferation of human keratinocytes through activation of NF-κB signaling (43). Neutrophils stimulated by monosodium urate delay the activation of transcription factors NF-κB and C/EBP and promote NET formation (44). Furthermore, NETs stimulate airway epithelial cells to express CXCL1, CXCL2 and CXCL8 through the TLR4/NF-κB pathway, thereby recruiting neutrophils to sites of inflammation (45). Yang et al. (46) demonstrated that decreased levels of miR-4512 in monocytes and macrophages from systemic lupus erythematosus patients promoted innate immune activation and neutrophil NET formation by targeting TLR4 and CXCL2. Park et al. (47) found that myeloid cells from severe patients with COVID-19 showed higher CXCL8 expression, which promoted neutrophil recruitment and recruited neutrophils highly expressed genes related to NETs. In addition, the presence of NETs in human solid tumors was found to be a considerable degree of individual variation, and circulating NETs show a positive correlated with IL-8 (48). IL-6 trans-signaling was strongly associated with NET formation induced by *Haemophilus influenzae* in chronic obstructive pulmonary disease (COPD) patients (49). Furthermore, there was a significant positive correlation between soluble IL-6 receptor and NET markers in bronchoalveolar lavage fluid from COPD patients (49). By performing LASSO regression on the DGF hub genes, we finally constructed a DGF prediction model based on 4-NRG (NFKBIA, NFIL3, SGK1 and CXCL8). The two groups of patients (high and low risk groups) stratified by this model differed significantly in the occurrence of postoperative DGF, the activity of inflammation-related signaling pathways, and the abundance of immune cell infiltration. Notably, our 4-NRG model showed better predictive power compared to other existing DGF prediction tools, including Kdri, Schold score and Irish score.

The results of functional enrichment analysis showed that allograft rejection pathway was significantly enriched in both the C2 and high-risk groups of patients. The occurrence of DGF after transplantation is closely related to the occurrence of allograft rejection and long-term graft failure. In order to effectively predict the survival of long-term graft and the possibility of allograft rejection, we also constructed a 2-NRG (CXCL8 and CXCL2) prognostic model for long-term transplant kidney outcome based on DGF hub genes. CXCL2 and CXCL8 included in this model are a class of chemokines that cause neutrophil recruitment (50). In malignant pleural effusions, methotrexate-packaging tumor cell-derived microparticles induces the recruitment of neutrophils to the pleural cavity *via* CXCL1 and CXCL2 released from macrophages, followed by recruited neutrophils are activated and release reactive oxygen species and NETs to kill tumor cells (51). CXCL8 can trigger neutrophils to produce NETs (11), and NETs can also activate IL-8 expression in human bronchial epithelial cells (52). In severe COVID-19 patients, systemic and neutrophil autocrine CXCL8 positive feedback loops initiate neutrophil activation, degranulation and NET formation, which exacerbate neutrophil-driven immunopathology (53). Our results also showed that high-risk recipients are more likely to develop allograft rejection after renal transplantation. As for the two NRGs included in our signature, CXCL8 is crucial negative determinant for islet survival after transplantation (54). Studies have confirmed the significant upregulation of CXCL8 in chronic antibody-mediated rejection after renal transplantation (55). In addition, in a mouse model of liver transplant rejection, CXCL2 is significantly elevated in serum, which is the gene most closely related to the functions of neutrophils (56).

To further confirm the reliability of the model, we performed experimental validation of CXCL8, a gene included in both models, to explore its role in renal IRI and NETs through constructing mouse IRI model. The result of qRT-PCR showed that Cxcl1 was significantly overexpressed in mouse kidney tissues after IRI. Pretreatment of G31P, an antagonist of CXCL8 (57), significantly alleviated renal IRI. In addition, G31P could also reduce the necrosis of renal tubular epithelial cells, inflammatory response and NET formation.

However, this research still has some limitations. First, more datasets are needed to further validate the stability of the prediction model, and in the future, with the further improvement of information on renal transplant patients, we may construct a more accurate nomogram for prognosis prediction by integrating our models with other information (such as clinical parameters). Second, with the in-depth study of NETs, the construction of NET-related gene sets needs to be further improved. Finally, clinical samples can be applied to validate the expression of NRGs in renal IRI. It is necessary to further explore the cellular and molecular mechanisms of NRGs in regulating NET formation. An in-depth understanding of the molecular mechanisms of NET formation could help us inhibit NETs *via* targeted drugs, and then attenuate renal IRI. Thus, such research could pave the way for new diagnostic and therapeutic strategies for managing renal IRI.

## Data availability statement

The original contributions presented in the study are included in the article/**Supplementary Material**. Further inquiries can be directed to the corresponding authors.

## Ethics statement

The animal experiment was reviewed and approved by the Ethics Committee of Beijing MDKN Biotechnology Co., Ltd. (Approval No: MDKN-2022-012).

## Author contributions

JW, FZ and XZ contributed equally to this work and should be listed as first co-authors. JW, FZ and XZ conceived, designed and wrote the manuscript. JZ, PC, ZS and WW revised the manuscript. ZS and WW supervised the manuscript. All authors contributed to the article and approved the submitted version.

## Acknowledgments

We would like to express our appreciation to GEO for providing the open-access databases utilized in this research study.

## Conflict of interest

The authors declare that the research was conducted in the absence of any commercial or financial relationships that could be construed as a potential conflict of interest.

## Publisher’s note

All claims expressed in this article are solely those of the authors and do not necessarily represent those of their affiliated organizations, or those of the publisher, the editors and the reviewers. Any product that may be evaluated in this article, or claim that may be made by its manufacturer, is not guaranteed or endorsed by the publisher.
