# Supplementary material for: Identification of renal ischemia reperfusion injury subtypes and predictive strategies for delayed graft function and graft survival based on neutrophil extracellular trap-related genes
Source: Front Immunol. 2022 Dec 1;13:1047367. doi: 10.3389/fimmu.2022.1047367 (PMC9752097; doi:10.3389/fimmu.2022.1047367)
Supplement: Supplementary file 1 [file Table_1.docx]

**Table S1:** Information of 137 neutrophil extracellular traps related genes (NRGs) collected in this research.

| **Gene symbol** | **Entrez Gene** | **Ensembl** | **Description** **(coding protein)** |
| --- | --- | --- | --- |
| SGK1 | 6446 | ENSG00000118515 | Serum/glucocorticoid regulated kinase 1 |
| ACTB | 60 | ENSG00000075624 | Actin beta |
| ACTG1 | 71 | ENSG00000184009 | Actin gamma 1 |
| ACTN1 | 87 | ENSG00000072110 | Actinin alpha 1 |
| ACTN4 | 81 | ENSG00000130402 | Actinin alpha 4 |
| AKT1 | 207 | ENSG00000142208 | AKT serine/threonine kinase 1 |
| AKT2 | 208 | ENSG00000105221 | AKT serine/threonine kinase 2 |
| ARPIN | 348110 | ENSG00000242498 | Actin related protein 2/3 complex inhibitor |
| ATG7 | 10533 | ENSG00000197548 | Autophagy related 7 |
| AZU1 | 566 | ENSG00000172232 | Azurocidin 1 |
| C3 | 718 | ENSG00000125730 | Complement C3 |
| C3AR1 | 719 | ENSG00000171860 | Complement C3a receptor 1 |
| C5AR1 | 728 | ENSG00000197405 | Complement C5a receptor 1 |
| CAMP | 820 | ENSG00000164047 | Cathelicidin antimicrobial peptide |
| CARD11 | 84433 | ENSG00000198286 | Caspase recruitment domain family member 11 |
| CASP1 | 834 | ENSG00000137752 | Caspase 1 |
| CAT | 847 | ENSG00000121691 | Catalase |
| CCDC25 | 55246 | ENSG00000147419 | Coiled-coil domain containing 25 |
| CCL2 | 6347 | ENSG00000108691 | C-C motif chemokine ligand 2 |
| CCL3 | 6348 | ENSG00000277632 | C-C motif chemokine ligand 3 |
| CCL4 | 6351 | ENSG00000275302 | C-C motif chemokine ligand 4 |
| CCL5 | 6352 | ENSG00000271503 | C-C motif chemokine ligand 5 |
| CD177 | 57126 | ENSG00000204936 | CD177 molecule |
| CD274 | 29126 | ENSG00000120217 | Programmed cell death 1 ligand 1 |
| CD44 | 960 | ENSG00000026508 | CD44 molecule (Indian blood group) |
| CEBPB | 1051 | ENSG00000172216 | CCAAT enhancer binding protein beta |
| CFTR | 1080 | ENSG00000001626 | ATP-binding cassette sub-family C, member 7 |
| CLEC4E | 26253 | ENSG00000166523 | Macrophage-inducible C-type lectin |
| CLEC6A | 93978 | ENSG00000205846 | Dectin-2 |
| CLEC7A | 64581 | ENSG00000172243 | Dectin-1 |
| CSF3 | 1440 | ENSG00000108342 | Granulocyte colony stimulating factor |
| CTSC | 1075 | ENSG00000109861 | Cathepsin C |
| CTSG | 1511 | ENSG00000100448 | Cathepsin G |
| CXCL1 | 2919 | ENSG00000163739 | C-X-C motif chemokine ligand 1 |
| CXCL2 | 2920 | ENSG00000081041 | C-X-C motif chemokine ligand 2 |
| CXCR4 | 7852 | ENSG00000121966 | C-X-C motif chemokine receptor 4 |
| CYBB | 1536 | ENSG00000165168 | NADPH oxidase |
| DEFA3 | 1668 | ENSG00000239839 | Defensin alpha 3 |
| DNAJB1 | 3337 | ENSG00000132002 | DnaJ heat shock protein family (Hsp40) member B1 |
| DNASE1 | 1773 | ENSG00000213938 | Deoxyribonuclease I |
| ELANE | 1991 | ENSG00000197561 | Neutrophil elastase |
| ENO1 | 2023 | ENSG00000074800 | Enolase 1 |
| ENTPD4 | 14573 | ENSG00000197217 | Ectonucleoside Triphosphate Diphosphohydrolase 4 |
| F2RL2 | 2151 | ENSG00000164220 | Proteinase-activated receptor-3 |
| F3 | 2152 | ENSG00000117525 | Coagulation Factor III, tissue factor |
| FCAR | 2204 | ENSG00000186431 | Fc fragment of IgA receptor |
| FCGR2B | 2213 | ENSG00000072694 | Fc gamma receptor IIb |
| FGL2 | 10875 | ENSG00000127951 | Fibrinogen like 2 |
| GPBAR1 | 151306 | ENSG00000179921 | G protein-coupled bile acid receptor 1 |
| GSDMD | 79792 | ENSG00000104518 | Gasdermin D |
| H2AX | 3014 | ENSG00000188486 | H2A histone family, member X |
| HIF1A | 3091 | ENSG00000100644 | Hypoxia inducible factor 1 subunit alpha |
| HMGB1 | 3146 | ENSG00000189403 | High mobility group box 1 |
| HRG | 3273 | ENSG00000113905 | Histidine rich glycoprotein |
| IL12A | 3592 | ENSG00000168811 | Interleukin 12A |
| IL17A | 3605 | ENSG00000112115 | Interleukin 17 |
| IL1B | 3553 | ENSG00000125538 | Interleukin 1 beta |
| IL1RL1 | 9173 | ENSG00000115602 | Interleukin 1 receptor like 1 |
| IL33 | 90865 | ENSG00000137033 | Interleukin 33 |
| IL36RN | 26525 | ENSG00000136695 | Interleukin 36 receptor antagonist |
| IL5 | 3567 | ENSG00000113525 | Interleukin 5 |
| IL6 | 3569 | ENSG00000136244 | Interleukin 6 |
| IL8 | 3576 | ENSG00000169429 | Interleukin 8 |
| ILK | 3611 | ENSG00000166333 | Integrin linked kinase |
| IRAK4 | 51135 | ENSG00000198001 | Interleukin 1 receptor associated kinase 4 |
| IRF1 | 3659 | ENSG00000125347 | Interferon regulatory factor 1 |
| ITGAM | 3684 | ENSG00000169896 | Complement component 3 receptor 3 subunit |
| ITGB1 | 3688 | ENSG00000150093 | Integrin subunit beta 1 |
| ITGB2 | 3689 | ENSG00000160255 | Complement component 3 receptor 3 and 4 subunit |
| KCNN3 | 3782 | ENSG00000143603 | Potassium channel, calcium activated |
| KLF2 | 10365 | ENSG00000127528 | KLF transcription factor 2 |
| KRT10 | 3858 | ENSG00000186395 | Keratin 10 |
| LCP1 | 3936 | ENSG00000136167 | Lymphocyte cytosolic protein 1 |
| LDLR | 3949 | ENSG00000130164 | Low density lipoprotein receptor |
| LPAR3 | 23566 | ENSG00000171517 | Lysophosphatidic acid receptor 3 |
| LTF | 4057 | ENSG00000012223 | Lactotransferrin |
| LYZ | 4069 | ENSG00000090382 | Lysozyme |
| MAPK1 | 5594 | ENSG00000100030 | Mitogen-activated protein kinase 1 |
| MAPK14 | 1432 | ENSG00000112062 | Mitogen-activated protein kinase 14 |
| MAPK3 | 5595 | ENSG00000102882 | Mitogen-activated protein kinase 3 |
| MAPK7 | 5598 | ENSG00000166484 | Mitogen-activated protein kinase 7 |
| MCOLN3 | 55283 | ENSG00000055732 | Mucolipin TRP cation channel 3 |
| MFN1 | 55669 | ENSG00000171109 | Mitofusin 1 |
| MFN2 | 9927 | ENSG00000116688 | Mitofusin 2 |
| MIR146A | 406938 | ENSG00000283733 | MicroRNA 146a |
| MIR21 | 406991 | ENSG00000284190 | MicroRNA 21 |
| MIR223 | 407008 | ENSG00000284567 | MicroRNA 223 |
| MMP9 | 4218 | ENSG00000100985 | Matrix metallopeptidase 9 |
| MNDA | 4332 | ENSG00000163563 | Myeloid cell nuclear differentiation antigen |
| MPO | 4353 | ENSG00000005381 | Myeloperoxidase |
| MTOR | 2475 | ENSG00000198793 | Mechanistic target of rapamycin kinase |
| MYD88 | 4615 | ENSG00000172936 | MYD88 innate immune signal transduction adaptor |
| MYH9 | 4627 | ENSG00000100345 | Myosin heavy chain 9 |
| NFE2L2 | 4780 | ENSG00000116044 | NFE2 like bZIP transcription factor 2 |
| NFIL3 | 4783 | ENSG00000165030 | Nuclear factor, interleukin 3 regulated |
| NFKBIA | 4792 | ENSG00000100906 | NFKB inhibitor alpha |
| NLRP3 | 114548 | ENSG00000162711 | NLR family pyrin domain containing 3 |
| NOX4 | 50507 | ENSG00000086991 | NADPH oxidase 4 |
| OPA1 | 4976 | ENSG00000198836 | OPA1 mitochondrial dynamin like GTPase |
| ORAI1 | 84876 | ENSG00000276045 | ORAI calcium release-activated calcium modulator 1 |
| P2RX1 | 5023 | ENSG00000108405 | Purinergic receptor P2X 1 |
| PADI4 | 23569 | ENSG00000159339 | Peptidyl arginine deiminase 4 |
| PARVB | 29780 | ENSG00000188677 | Parvin beta |
| PF4 | 5196 | ENSG00000163737 | Chemokine (C-X-C motif) ligand 4 |
| PIK3CA | 5290 | ENSG00000121879 | Phosphatidylinositol-4,5-bisphosphate 3-kinase |
| PKM | 5315 | ENSG00000067225 | Pyruvate kinase M1/2 |
| PROCR | 10544 | ENSG00000101000 | Protein C receptor |
| PRTN3 | 5657 | ENSG00000196415 | Proteinase 3 |
| PTAFR | 5724 | ENSG00000169403 | Platelet activation factor receptor |
| RIPK1 | 8737 | ENSG00000137275 | Receptor interacting serine/threonine kinase 1 |
| RIPK3 | 11035 | ENSG00000129465 | Receptor interacting serine/threonine kinase 3 |
| S100A12 | 6283 | ENSG00000163221 | S100 calcium binding protein 2 |
| S100A8 | 6279 | ENSG00000143546 | S100 calcium binding protein A8 |
| S100A9 | 6280 | ENSG00000163220 | S100 calcium binding protein A9 |
| S1PR2 | 9294 | ENSG00000267534 | Sphingosine-1-phosphate receptor 2 |
| SELP | 6403 | ENSG00000174175 | P-selectin |
| SELPLG | 6404 | ENSG00000110876 | P-selectin receptor |
| SGK1 | 6446 | ENSG00000118515 | Serum/glucocorticoid regulated kinase 1 |
| SIGLEC14 | 10049587 | ENSG00000254415 | Sialic acid binding Ig like lectin 4 |
| SOCS3 | 9021 | ENSG00000184557 | Suppressor of cytokine signaling 3 |
| SPP1 | 6696 | ENSG00000118785 | Secreted phosphoprotein 1 |
| SRC | 6714 | ENSG00000197122 | SRC proto-oncogene, non-receptor tyrosine kinase |
| STAT3 | 6774 | ENSG00000168610 | Signal transducer and activator of transcription 3 |
| SUCNR1 | 56670 | ENSG00000198829 | Succinate receptor 1 |
| SYK | 6850 | ENSG00000165025 | Spleen associated tyrosine kinase |
| TICAM1 | 148022 | ENSG00000127666 | TIR domain-containing adaptor-inducing interferon-beta |
| TIMP1 | 7076 | ENSG00000102265 | TIMP metallopeptidase inhibitor 1 |
| TKT | 7086 | ENSG00000163931 | Transketolase |
| TLR2 | 7097 | ENSG00000137462 | Toll like receptor 2 |
| TLR4 | 7099 | ENSG00000136869 | Toll like receptor 4 |
| TLR7 | 51284 | ENSG00000196664 | Toll like receptor 7 |
| TLR8 | 51311 | ENSG00000101916 | Toll like receptor 8 |
| TLR9 | 54106 | ENSG00000239732 | Toll like receptor 9 |
| TNF | 7124 | ENSG00000232810 | Tumor necrosis factor-alpha |
| TNFAIP3 | 7128 | ENSG00000118503 | TNF alpha induced protein 3 |
| WASL | 8976 | ENSG00000106299 | WASP like actin nucleation promoting factor |
| XIST | 7503 | ENSG00000229807 | X inactive specific transcript |
