# Supplementary material for: Identification of renal ischemia reperfusion injury subtypes and predictive strategies for delayed graft function and graft survival based on neutrophil extracellular trap-related genes
Source: Front Immunol. 2022 Dec 1;13:1047367. doi: 10.3389/fimmu.2022.1047367 (PMC9752097; doi:10.3389/fimmu.2022.1047367)
Supplement: Supplementary file 2 [file Table_2.docx]

**Table S2:** Differentially expressed neutrophil extracellular trap-related genes (DE-NRGs) with absolute log_2_ fold change (FC) > 0.5 and FDR < 0.05.

| **DE-NRGs** | **Log_2_ FC** | **P Value** | **FDR** |
| --- | --- | --- | --- |
| IRF1 | 1.45214067 | 2.38E-42 | 7.39E-40 |
| LDLR | 1.37085366 | 1.54E-33 | 3.00E-31 |
| DNAJB1 | 2.64189273 | 2.80E-59 | 5.48E-56 |
| KLF2 | 2.34856238 | 5.64E-63 | 2.78E-59 |
| SGK1 | 0.61953449 | 1.14E-15 | 8.65E-14 |
| CXCL8 | 1.13457636 | 2.48E-43 | 8.66E-41 |
| CXCL2 | 1.61394967 | 5.26E-56 | 5.58E-53 |
| NFKBIA | 1.02108696 | 3.72E-39 | 1.01E-36 |
| CEBPB | 1.04333932 | 4.01E-33 | 7.55E-31 |
| CCL3 | 0.77344101 | 6.14E-43 | 2.03E-40 |
| SOCS3 | 0.88455674 | 1.48E-48 | 7.37E-46 |
| CXCL1 | 0.69304072 | 7.76E-36 | 1.78E-33 |
| NFIL3 | 1.07899312 | 6.54E-44 | 2.48E-41 |
| TNFAIP3 | 0.93864745 | 8.96E-52 | 5.84E-49 |
| CCL2 | 0.96950752 | 2.60E-14 | 1.77E-12 |
| IL6 | 0.61928529 | 2.49E-32 | 4.53E-30 |
