# Supplementary material for: Identification of renal ischemia reperfusion injury subtypes and predictive strategies for delayed graft function and graft survival based on neutrophil extracellular trap-related genes
Source: Front Immunol. 2022 Dec 1;13:1047367. doi: 10.3389/fimmu.2022.1047367 (PMC9752097; doi:10.3389/fimmu.2022.1047367)
Supplement: Supplementary file 3 [file Table_3.docx]

**Table S3**: Feature genes selected by random forest (RF) algorithm and support vector machine recursive feature elimination (SVM-RFE) algorithm.

| **RF** | **SVM-RFE** |
| --- | --- |
| SGK1 | SGK1 |
| NFKBIA | NFKBIA |
| DNAJB1 | DNAJB1 |
| CXCL8 | NFIL3 |
| NFIL3 | CXCL8 |
| IL6 | IL6 |
| CXCL2 | CEBPB |
| SOCS3 | CXCL2 |
| CEBPB | CXCL1 |
| CCL2 |  |
