# Supplementary material for: Identification of renal ischemia reperfusion injury subtypes and predictive strategies for delayed graft function and graft survival based on neutrophil extracellular trap-related genes
Source: Front Immunol. 2022 Dec 1;13:1047367. doi: 10.3389/fimmu.2022.1047367 (PMC9752097; doi:10.3389/fimmu.2022.1047367)
Supplement: Supplementary file 4 [file Table_4.docx]

|  | Sensitivity | Specificity | AUC |
| --- | --- | --- | --- |
| Fold 1 | 0.82 | 0.78 | 0.80 |
| Fold 2 | 0.91 | 0.78 | 0.84 |
| Fold 3 | 0.73 | 0.89 | 0.81 |
| Fold 4 | 1 | 0.89 | 0.95 |
| Fold 5 | 0.83 | 0.4 | 0.62 |
| Fold 6 | 0.73 | 0.67 | 0.70 |
| Fold 7 | 0.73 | 0.78 | 0.75 |
| Fold 8 | 0.82 | 0.56 | 0.69 |
| Fold 9 | 0.91 | 0.67 | 0.79 |
| Fold 10 | 0.82 | 0.33 | 0.58 |
| Mean | 0.83 | 0.67 | 0.75 |
| Min | 0.73 | 0.33 | 0.58 |
| Median | 0.82 | 0.72 | 0.77 |
| Max | 1 | 0.89 | 0.95 |

Table S4: 10-fold cross-validation of the model in the whole GSE43974 dataset.
